# Association between shift work and poor sleep quality in an Asian multi-ethnic working population: A cross-sectional study

**DOI:** 10.1371/journal.pone.0229693

**Published:** 2020-03-04

**Authors:** Thuan-Quoc Thach, Dhiya Mahirah, Gerard Dunleavy, Yichi Zhang, Nuraini Nazeha, Yuri Rykov, Audrey Nah, Adam Charles Roberts, George I. Christopoulos, Chee-Kiong Soh, Josip Car

**Affiliations:** 1 Centre for Population Health Sciences, Lee Kong Chian School of Medicine, Nanyang Technological University, Singapore, Singapore; 2 School of Mechanical and Aerospace Engineering, Nanyang Technological University, Singapore, Singapore; 3 Division of Strategy, Management and Organisation, Nanyang Business School, College of Business, Nanyang Technological University, Singapore, Singapore; 4 School of Civil and Environmental Engineering, College of Engineering, Nanyang Technological University, Singapore, Singapore; Universitat de Valencia, SPAIN

## Abstract

**Background:**

We aimed to examine the association between shift work and sleep quality in a diverse occupational type.

**Methods:**

This was a cross-sectional study of self-reported sleep quality in 424 workers aged ≥21 using the Pittsburgh Sleep Quality Index (PSQI). We divided workers into two categories based on their PSQI score: (a) ≤5 (good sleep quality) and (b) >5 (poor sleep quality). We used multiple logistic regressions to assess the association between shift work and sleep quality adjusted for potential confounders.

**Results:**

The mean age was 39.2 (SD = 11.3) years, with shift workers being older than their counterparts. Most workers were of Chinese ethnicity (63.9%). Males were significantly more likely to undertake shift work than females (89% *v* 11%, p-value<0.001), but it should be noted that the majority of workers was male (78.8%) in this sample of workers. Shift workers had a 198% increased odds of poor sleep quality compared to non-shift workers (OR = 2.98; 95% CI:1.53–5.81).

**Conclusion:**

Shift work was significantly and independently associated with increased odds of poor sleep quality in this sample of workers. The present findings may inform employment guidelines and help develop workplace health promotion interventions aimed at improving sleep quality among workers and ultimately lead to a healthier workforce.

## Introduction

Shift work is generally taken to mean any arrangement of daily working hours other than the standard daylight hours (7–8 am to 5–6 pm) [1]. Shift work is prevalent throughout the world. In the United States, according to the Bureau of Labor Statistics, it is estimated that 18% of full-time salaried workers are employed working alternate shifts [2]. Similar prevalence has been reported from Europe and Japan [3, 4]. In addition, shift work has been thought to have important health implications, with evidence linking shift work to an increased risk of cardiovascular disease, diabetes, gastrointestinal problems, reproductive disorders, mental health disorders, psychological stress, poor quality of life and several types of cancers [5–13]. In 2007, the World Health Organization (WHO) classified night shift work as a potential carcinogen due to its disruption of the body's circadian rhythm. Several hypotheses have been proposed to explain the relationship between shift work and cardiovascular disease [14]. Sleep disturbance has been suggested as an important factor, notably poor sleep quality is considered an independent risk factor for cardiovascular disease [15]. Poor sleep quality is characterised by difficulty of falling and remaining asleep [16]. It has been shown to be more prevalent among shift workers [17, 18].

In Singapore, shift work has become an economic necessity in many local industries because of emphasis on capital-intensive industries and the growing demand for round the clock health, transportation, and food services. This has led to a concern regarding the potential disturbed sleep quality for the shift workers in these industries [19]. Poor sleep affects cognitive and physical functioning, and insomnia is associated with a greater risk of falls and accidents, [20] higher rates of absenteeism, [21] and increased health care utilization [21]. Most of the available reports on sleep disturbance focused on the general population of Singapore, [22, 23] in whom the problem may be different in magnitude, nature and aetiology. Studies conducted in the workplace environment are limited despite the rapidly growing participation rates of shift workers in the labour force [24]. To this end, we set out to examine the possible association between shift work and sleep quality using a large cross-sectional study [25].

## Methods

### Study design and setting

**Fig 1:**
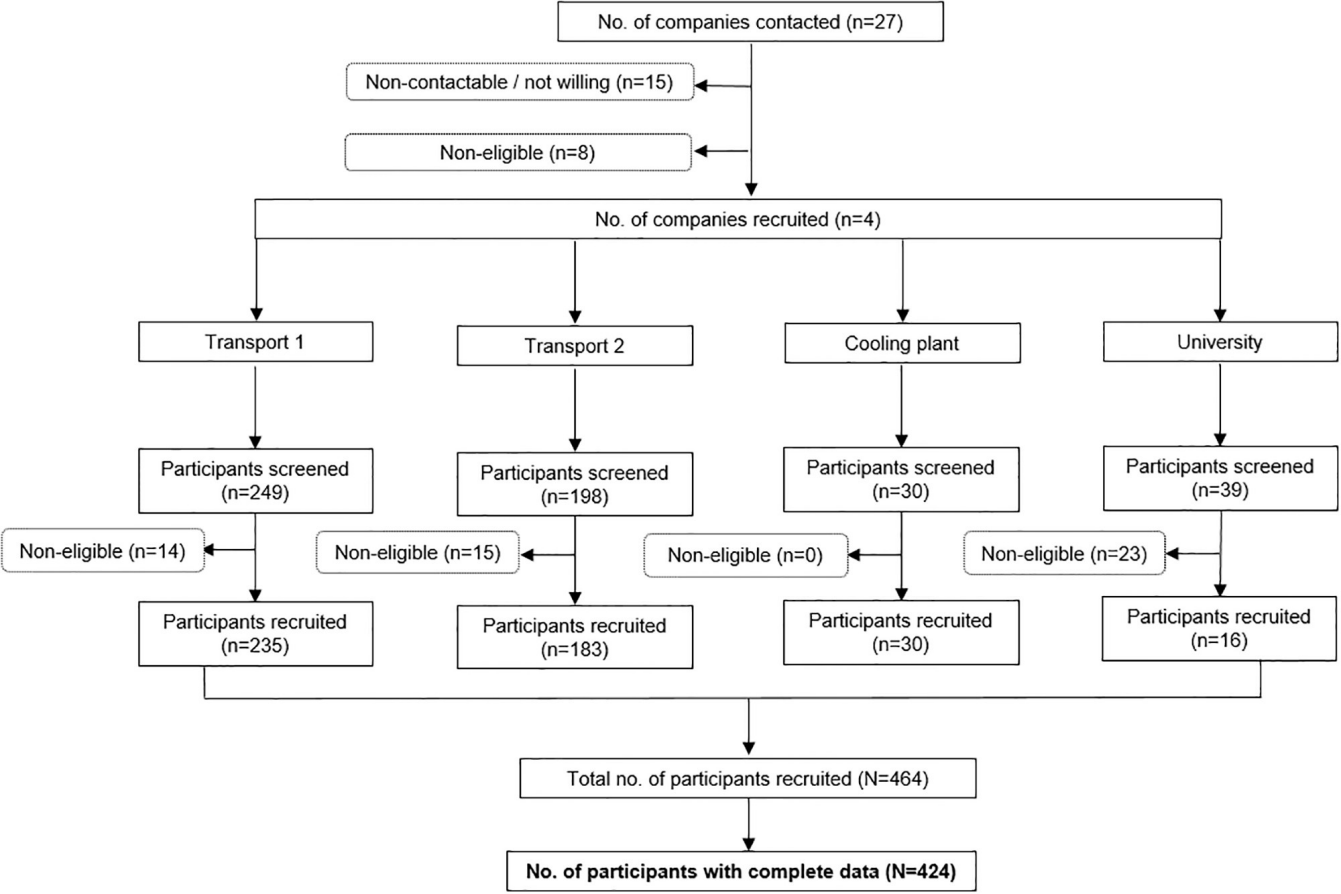
Flow diagram of recruitment process of study participants.

Through online searches and discussions with civil engineers who were part of our research team, we initially prepared a list of companies with underground workspaces including banks, transport sector, cooling plants, learning centres, mail service centres, hospitals, libraries, and universities. We then identified companies having aboveground workspaces with comparable job types as those with underground workspaces. In total, 27 companies were contacted through personal visits, phone calls and emails. Of which, 15 could not be reached or not willing, and eight were small with less than 20 employees. After excluding these, four companies were recruited, which included two transport industries, a cooling plant and a university. From these four companies, 10 work sites were chosen based on the estimated number of workers who would meet our criteria (e.g. sites were excluded if the workers did not stay in their office for most of their time), and workers in these sites were recruited in two steps. First, the study team approached the worksites and met with the senior management team to discuss the study. Once confirmation of participation from the management team was obtained, employees were invited to participate via worksite posters, meetings and emails. Employees expressed their interest through their management team or directly registered with the study team at the recruitment session. Those willing to participate were screened for eligibility. Participants were eligible for selection if they were aged ≥21 years who could speak English and worked for at least four hours a day at their assigned workspace, not pregnant, and had not made at least one trip per month on average to countries in a different time zone from Singapore in the past six months. A total of 516 workers were screened, of whom 464 were eligible, and recruited into the study. There were 424 completed data available for analysis. The total of number of workers in all companies was 2109 and the participation rate was 20%. Data collection for this study was conducted from August 2017 to March 2018. A summary of the recruitment process of study participants is shown in Fig 1.

### Outcome

The primary outcome variable of this study, sleep quality in the last month, was assessed according to the Pittsburgh Sleep Quality Index, PSQI, [26] a reliable and valid instrument to screen for any sleep disorders. It is composed of 19 items to assess seven components of sleep. Each component is scored from 0 to 3 points, where lower score denotes better sleep, while higher score denotes worsening problems in the following order: (1) subjective sleep quality (very good to very bad), (2) sleep latency (≤15 min to >60 min), (3) sleep duration (≥7 hours to <5 hours), (4) sleep efficiency (≥85% to <65% hours sleep/hours in bed), (5) sleep disturbances (not during the past month to ≥3 times per week), (6) use of sleeping medications (none to ≥3 times a week), and (7) daytime dysfunction (not a problem to a very big problem). All seven components are then summed up to produce a composite score from 0 to 21, with higher scores reflecting worse sleep quality. In this study, we dichotomised workers into two categories based on the composite score of PSQI: (a) ≤5 (good sleep quality) *vs* (b) >5 (poor sleep quality). We used the three-factor model of the PSQI which was previously reported in the Singapore general population to identify factors of the PSQI associated with shift work [27]. Factor 1 (perceived sleep quality) is formed by summing the components of (i) subjective sleep quality and (ii) sleep latency; Factor 2 (sleep efficiency) the components of (iii) sleep duration and (iv) sleep efficiency; and Factor 3 (daily disturbances) the components of (v) sleep disturbances, (vi) sleeping medication, and (vii) daytime dysfunction.

### Shift work

Workers were classified as shift workers if they answered “yes” to the question “Do you currently work in shifts?” and then provided information on shift work hours per day.

### Covariates

The following covariates were included in the analysis as potential confounders in the association between shift workers and sleep quality. The categorisation of adjustment confounders was specified *a priori*. The potential confounders were considered in the analysis on the basis a literature review to identify factors previously shown to be related to sleep quality and shift work [28–31].

### Socio-demographic data

Self-administered questionnaires were used to collect data on socio-demographic characteristics, including age (years), gender (male or female), educational level (primary/secondary, pre-college, and college and above), marital status (single/never married/widowed/divorced and married) and ethnicity (Chinese, Malay, Indian or others).

### Lifestyle and health behaviour data

Components of lifestyle and health-related behaviours were collected via self-reported questionnaires including current smoking status (yes or no), comorbidity (yes: at least 1 morbidity; no: no morbidity) and alcohol consumption in the last 12 months (yes or no). Caffeine intake (servings/day) was estimated from coffee, tea and soda consumption reported in a Food Frequency Questionnaire adapted from the FFQ used in Singapore’s National Nutrition Survey [32]. Regarding the level of physical activity of workers, we employed the Global Physical Activity Questionnaire (GPAQ), which measures activity levels in three domains namely, work, travel, and leisure [33]. A Metabolic Equivalent (MET) value of four was assigned for moderate physical activities and a MET value of eight for vigorous physical activities. The duration (in minutes) of an activity performed in each of the three domains was multiplied by its MET value, and these were summed to obtain an estimate of the total MET-min/week. Workers with total MET-min/week <600, 600–2999 and ≥3000 were classified as less active, moderately active, and highly active, respectively. We used the General Health Questionnaire-12 (GHQ-12) to assess the psychological distress of workers [34]. The GHQ-12 comprises items capturing symptoms of depression and anxiety over the previous four weeks. Item response is based on a 4-point scale that indicates the presence of a symptom (“not at all” = 0, “same as usual” = 0, “more than usual” = 1, “much more than usual” = 1). Points for each item were summed to give a GHQ-12 global score that ranges from 0 to 12. It provides a count of the number of positive responses indicative of psychological distress. We also measured Body Mass Index (BMI) which is derived from the ratio of weight in kilograms to height in metres squared.

### Work-related characteristics

In addition to the above covariates, the study asked participants about their occupation and number of hours worked per day. Work type was aggregated into three distinct groups: office workers, control room workers, and workshop workers. Participants were also categorised according to type of industry: transport, energy and university.

### Statistical analysis

Student-t, Wilcoxon rank-sum, and Chi-square tests were used to evaluate the sample mean or median differences in continuous and categorical variables, respectively.

We performed a multiple logistic regression model to examine the extent to which shift work is associated with poor sleep quality. Analyses were adjusted for socio-demographic characteristics: lifestyle and health-related behaviour and work-related factors.

The analysis was conducted in three hierarchical stages. First, the model was built using socio-demographic variables as the predictors (Model 1). Second, variables representing lifestyle and health-related behaviours were entered (Model 2). Finally, work-related variables were entered into the model (Model 3). In all the models, the comparison group of this study was non-shift workers.

Results of the multiple logistic regression models were expressed as adjusted odds ratios (OR) of poor sleep quality associated with shift workers, with 95% confidence intervals (CI) using robust standard errors.

All statistical tests were two sided; p-values <0.05 were considered statistically significant. All analyses were performed using Stata Version 15.0 (StataCorp, College Station, TX, USA) and R Version 3.4.4 (R Foundation for Statistical Computing, Vienna, Austria).

### Ethics approval

The study was approved by the Institutional Review Board of Nanyang Technological University (NTU) (IRB-2015-11-028). Study participants provided written informed consent prior to the commencement of data collection.

## Results

### General characteristics of the study population

Table 1 summarizes the characteristics of shift and non-shift workers. The mean age was 39.2 (SD = 11.3) years, with shift workers being older than their counterparts. Most workers were of Chinese ethnicity (63.9%). Males were significantly more likely to undertake shift work than females (89% *v* 11%, p-value<0.001), but it should be noted that the majority of workers was male (78.8%) in this sample of workers.

**Table 1:** Characteristics of study participants stratified by shift work (N = 424).

	Total N = 424		Shift workers N = 155 (36.6%)		Non-shift workers N = 269 (63.4%)		P-value*
	n	%	n	%	n	%	
**Socio-demographic**							
Age (years)^d^	39.2	(11.3)	39.9	11.3	38.7	(11.3)	0.232
Gender							<0.001
Male	334	78.8	138	89.0	196	72.9	
Female	90	21.2	17	11.0	73	27.1	
Education level							<0.001
Primary & secondary	40	9.4	15	9.7	25	9.3	
Pre-college	234	55.2	107	69.0	127	47.2	
College & above	150	35.4	33	21.3	117	43.5	
Marital status							0.619
Single^a^	168	39.6	59	38.1	109	40.5	
Married	256	60.4	96	61.9	160	59.5	
Ethnicity							0.019
Chinese	271	63.9	93	60.0	178	66.2	
Malay	89	21.0	43	27.7	46	17.1	
Indian	44	10.4	16	10.3	28	10.4	
Others^b^	20	4.7	3	1.9	17	6.3	
**Health / lifestyle behaviour**							
Smoking status							0.001
No	277	65.3	86	55.5	191	71.0	
Yes	147	34.7	69	44.5	78	29.0	
Sleep Quality							<0.001
Good sleep	241	56.8	70	45.2	171	63.6	
Poor sleep	183	43.2	85	54.8	98	36.4	
Comorbidity							0.280
No	276	65.1	106	68.4	170	63.2	
Yes	148	34.9	49	31.6	99	36.8	
Alcohol consumption							0.639
No	207	48.8	78	50.3	129	48.0	
Yes	217	51.2	77	49.7	140	52.0	
Physical activity level^c^							0.557
Low (<600)	126	29.7	49	31.6	77	28.6	
Moderate (600–2999)	187	44.1	70	45.2	117	43.5	
High (≥3000)	111	26.2	36	23.2	75	27.9	
GHQ-12 global score ^d^	0.0	[2.0]	0.0	[2.0]	0.0	[2.0]	0.400
Caffeine intake^d^ (serving/day)	1.4	[1.3]	2.0	[1.3]	1.3	[1.4]	0.009
Body mass index ^d^	24.7	[6.0]	25.6	[6.2]	24.4	[5.3]	0.016
**Work-related factors**							
Occupation type							<0.001
Office worker	201	47.4	12	7.7	189	70.3	
Control room worker	131	30.9	117	75.5	14	5.2	
Workshop worker	92	21.7	26	16.8	66	24.5	
Industry type							
Energy	30	7.1	26	16.8	4	1.5	<0.001
Transport	379	89.4	129	83.2	250	92.9	
University	15	3.5	0	0.0	15	5.6	
Working hours per day^d^	8.3	[1.0]	8.3	[1.0]	8.5	[1.0]	0.353

GHQ: General Health Questionnaire

*P-value obtained from Chi-squared test for categorical variable and Wilcoxon rank-sum test for non-normally distributed continuous variable

^a^ Included never married, widowed and divorced

^b^ Included mixed ethnicities: Indonesians, Pakistanis, and Filipinos

^c^ MET-min/per week: Metabolic equivalent value minute per week

^d^ Mean and standard deviation (SD) reported for continuous variable, median and inter-quartile range [IQR] reported for non-normally distributed continuous variable

Shift workers had significantly higher prevalence of smoking (p-value = 0.001), poor sleep quality (p-value<0.001), higher BMI (p-value = 0.016) and consumed significantly more caffeine beverage (p-value = 0.009) than non-shift workers. Shift workers tended to perform lower levels of highly active physical activity (≥3000 MET-min/week) than their counterparts (23.2% *v* 27.9%). There were significant differences in the prevalence of shift and non-shift workers among the three types of occupations (p-value<0.001). In addition, there were significant differences in the prevalence of shift and non-shift workers among the three types of industry (p-value <0.001). No significant difference in working hours was found between the two shifts of work.

Table 2 presents the prevalence of sleep quality according to the characteristics of study participants. On average, workers with poor sleep quality were older than those with good sleep quality (40.2 years *v* 38.4 years, p = 0.099). There was a significantly higher prevalence of poor sleep quality in shift workers compared to non-shift workers (54.8%; 95% CI: 46.7%-62.8% *v* 36.4%; 95% CI: 30.7%-42.4%; p-value<0.001). In general, workers with poor sleep quality tended to have lower educational attainment (Primary & secondary: 62.5% *v* 37.5; Pre-college: 45.3% *v* 54.7%; College & above: 34.7% *v* 65.3%, p-value = 0.004), less likely to perform physical activity, scored higher on the GHQ-12 indicating increased psychological distress (1.0 *v* 0.0, p-value<0.001), and consumed more caffeine beverages than those with good sleep quality (2 serving/day *v* 1.3 serving/day, p-value = 0.005). No significant differences in the number of hours worked per day between the two qualities of sleep were observed.

**Table 2:** Prevalence sleep quality by characteristics of study participants (N = 424).

	PSQI: Sleep quality				P-value
	> 5 (Poor) N = 183 (43.2%)		≤ 5 (Good) N = 241(56.8%)		
	n	%	n	%	
**Socio-demographics**					
Age (years)^d^	40.2	(11.4)	38.4	(11.2)	0.099
Gender					0.495
Male	147	44.0	187	56.0	
Female	36	40.0	54	60.0	
Education level					0.004
Primary & secondary	25	62.5	15	37.5	
Pre-college	106	45.3	128	54.7	
College & above	52	34.7	98	65.3	
Marital status					0.192
Single^a^	66	39.3	102	60.7	
Married	117	45.7	139	54.3	
Ethnicity					0.059
Chinese	111	41.0	160	59.0	
Malay	48	53.9	41	46.1	
Indian	14	31.8	30	68.2	
Others^b^	10	50.0	10	50.0	
**Health / lifestyle behaviour**					
Smoking status					0.177
No	113	40.8	164	59.2	
Yes	70	47.6	77	52.4	
Comorbidity					0.292
No	114	41.3	162	58.7	
Yes	69	46.6	79	53.4	
Alcohol consumption					0.394
No	85	41.1	122	58.9	
Yes	98	45.2	119	54.8	
Physical activity level^c^					0.340
Low (<600)	61	48.4	65	51.6	
Moderate (600–2999)	75	40.1	112	59.9	
High (≥3000)	47	42.3	64	57.7	
GHQ-12 global score ^d^	1.0	[2.0]	0.0	[1.0]	<0.001
Caffeine intake^d^ (serving/ day)	2.0	[1.4]	1.3	[1.4]	0.005
Body mass index ^d^	25.3	[5.4]	24.5	[6.4]	0.286
**Work-related factors**					
Shift work					<0.001
No	98	36.4	171	63.6	
Yes	85	54.8	70	45.2	
Occupation type					0.042
Office worker	74	36.8	127	63.2	
Control room worker	65	49.6	66	50.4	
Workshop worker	44	47.8	48	52.2	
Industry type					
Energy	17	56.7	13	43.3	0.063
Transport	163	43.0	216	57.0	
University	3	20.0	12	80.0	
Working hours per day^d^	8.3	[1.0]	8.3	[1.0]	0.342

PSQI: Pittsburgh Sleep Quality Index; GHQ: General Health Questionnaire

*P-value obtained from Chi-squared test for categorical variable and Wilcoxon rank-sum test for non-normally distributed continuous variable

^a^ Included never married, widowed and divorced

^b^ Included mixed ethnicities: Indonesians, Pakistanis, and Filipinos

^c^ MET-min/per week: Metabolic equivalent value minute per week

^d^ Mean and standard deviation (SD) reported for continuous variable, median and inter-quartile range [IQR] reported for non-normally distributed continuous variable

Further analyses of the PSQI factors and specific subscales were performed to compare differences in scores between shift and non-shift workers (Table 3). Scores for Factor 1: perceived sleep quality factor (p-value<0.001) and Factor 2: sleep efficiency (p-value<0.001) were significantly higher (poorer sleep quality) in shift workers as compared to non-shift workers. Similarly, significant differences in the components that formed these factors were observed: (i) subjective sleep quality (p-value = 0.006), (ii) sleep latency (p-value = 0.001), (iii) sleep duration (p-value = 0.038); (iv) and sleep efficiency (p-value = 0.001).

**Table 3:** Comparison of PSQI subscales and factors between shift and non-shift workers.

	Shift (n = 155)		Non-shift (n = 269)		P-value*
	Mean	95% CI	Mean	95% CI	
**Factor 1: Perceived sleep quality**	2.16	1.97–2.35	0.71	1.58–1.84	<0.001
(i) Subjective sleep quality	1.07	0.99–1.16	0.93	0.86–0.99	0.006
(ii) Sleep latency	1.09	0.95–1.23	0.78	0.86–0.99	0.001
**Factor 2: Sleep efficiency**	2.05	1.78–2.31	1.41	1.24–1.59	<0.001
(iii) Sleep duration	1.08	0.93–1.24	0.87	0.77–0.98	0.038
(iv) Sleep efficiency	0.96	0.77–1.15	0.54	0.43–0.65	0.001
**Factor 3: Daily disturbances**	1.94	1.76–2.12	1.89	1.74–2.03	0.357
(v) Sleep disturbances	1.12	1.02–1.21	1.08	1.01–1.14	0.483
(vi) Sleeping medication	0.08	0.03–0.13	0.10	0.04–0.16	0.469
(vii) Daytime dysfunction	0.74	0.64–0.85	0.71	0.62–0.79	0.426

PSQI: Pittsburgh Sleep Quality Index- higher values denote worse sleep quality, CI: Confidence interval

*P-value from Wilcoxon rank sum test

### Association between shift worker and sleep quality

Table 4 shows the odds ratios for poor sleep quality in shift workers adjusted for potential confounders. The salient point from this table is that irrespective of the adjustment for a range of potential confounders, there was a strong significant relationship between shift worker and sleep quality such that higher odds of poor sleep quality was observed in shift workers. In other words, shift work was significantly associated with increased odds of poor sleep quality.

**Table 4:** Odds ratios (OR) and 95% confidence intervals (CI) for poor sleep associated with shift work.

	Model 0		Model 1		Model 2		Model 3	
	OR	95% CI	OR	95% CI	OR	95% CI	OR	95% CI
**Shift work**								
No	1		1		1		1	0.001 ^d^
Yes	2.12	1.42–3.17**	2.04	1.33–3.13**	2.12	1.34–3.34**	2.98	1.53–5.81**
**Socio-demographic**								
Age (years)			1.01	0.98–1.03	1.01	0.98–1.03	1.01	0.98–1.04
Gender								
Male			1		1		1	0.597 ^d^
Female			1.00	0.59–1.69	1.12	0.63–1.99	1.18	0.64–2.17
Education level								
Primary & secondary			1		1		1	0.056 ^d^
Pre-college			0.47	0.22–1.01	0.37	0.16–0.88*	0.37	0.15–0.90
College & above			0.40	0.17–0.92*	0.26	0.10–0.68*	0.29	0.10–0.82
Marital status								
Single^a^			1		1		1	0.745 ^d^
Married			1.14	0.69–1.88	1.11	0.64–1.91	1.10	0.63–1.91
Ethnicity								
Chinese			1		1		1	0.008 ^d^
Malay			1.56	0.90–2.73	2.55	1.31–4.97*	2.70	1.34–5.42*
Indian			0.67	0.32–1.38	0.83	0.36–1.92	0.80	0.34–1.90
Others^b^			1.90	0.76–4.76	2.64	0.98–7.08	2.52	0.93–6.84
**Health / lifestyle factors**								
Smoking status								
No					1		1	0.544 ^d^
Yes					0.86	0.51–1.47	0.85	0.50–1.45
Comorbidity								
No					1		1	0.540
Yes					1.16	0.72–1.86	1.16	0.72–1.87
Alcohol consumption								
No					1		1	0.078 ^d^
Yes					1.48	0.91–2.42	1.57	0.95–2.57
Physical activity level^c^								
Low (<600)					1		1	0.421 ^d^
Moderate (600–2999)					0.79	0.47–1.32	0.80	0.48–1.33
High (≥3000)					0.68	0.38–1.22	0.67	0.37–1.22
GHQ-12 global score					1.31	1.16–1.48**	1.30	1.15–1.47**
Caffeine intake^d^ (serving/ day)					1.14	0.96–1.36	1.14	0.96–1.36
Body mass index					1.00	0.96–1.04	1.00	0.96–1.04
**Work-related factors**								
Occupation type								
Office worker							1	0.359 ^d^
Control room worker							0.59	0.28–1.27
Workshop worker							1.00	0.51–1.95
Industry type								
Energy							1	0.547 ^d^
Transport							0.83	0.26–2.63
University							0.41	0.07–2.50
Working hours per day							1.00	0.82–1.22

OR: odds ratio; CI: confidence interval

P-value:

** <0.001;

*<0.05

^a^ Includes never married, widowed and divorced

^b^ Includes mixed ethnicities: Indonesians, Pakistanis, and Filipinos

^c^ MET-min/per week: Metabolic equivalent value minute per week

^d^ Overall P-value for categorical variable

In the initial model (Model 0) with adjustment only for shift work, the OR for poor sleep quality in shift workers was 2.12 (95% CI: 1.42–3.17). Adjustment for socio-demographic factors (Model 1) attenuated the association between shift work and poor sleep, with OR = 2.04 (95% CI: 1.33–3.13). Adjustment for socio-demographics, lifestyle, and health factors (Model 2) increased OR to 2.12 (95% CI: 1.34–3.34). Finally, adjustment for all potential confounders (Model 3) further increased OR to 2.98 (95% CI: 1.53–5.81), suggesting a positive confounding. In addition, the OR for poor sleep quality associated with a 1-unit increase in GHQ-12 global score was 1.30 (95% CI: 1.15–1.47). Ethnicity was also associated with sleep quality (overall p-value = 0.008).

## Discussion

The aim of this cross-sectional study was to examine the possible association between shift work and sleep quality. There was a significantly higher prevalence of poor sleep quality in shift workers compared to non-shift workers (54.8% *v* 36.4%). Furthermore, based on the three-factor PSQI model, shift-workers had significantly higher scores on Factor 1: perceived sleep quality and Factor 2: sleep efficiency. Shift workers had significantly worse scores on the four subscales (sleep latency, sleep duration, sleep efficiency, and subjective sleep quality) which constitute these two factors. We also found that shift work was significantly associated with an increased odds of poor sleep quality after adjustment for potential confounders. Shift workers had a 198% increased odds of poor sleep quality compared to non-shift workers. The association remained robust after adjustment for age, gender, educational level, marital status, BMI, physical activity, alcohol consumption, caffeine intake, psychological distress, occupational type, industry type and work hours per day.

### Comparison with other studies

Our findings are in line with previous studies exploring the associations between shift work and sleep quality. Specifically, a number of cross-sectional studies conducted among police officers, and nurses showed shift workers had higher odds of poor sleep quality compared to non-shift workers [35, 36]. However, the magnitude of the effect is not directly comparable either in terms of the occupation type or the nature of shift work. Numerous studies conducted among a large sample of shift workers showed that they were more likely to report poor health, be overweight or obese, type 2 diabetes, decreased immunity, and depression [37–40].

Besides shift work, we report three additional significant factors associated with poor sleep quality, namely GHQ-12 scores and ethnicity. A rise in psychological distress was significantly and independently associated with poor sleep quality. This result is consistent with extant research in that mental health problems are a potential mediator linking shift work to poor sleep quality [41–43]. This suggests that mental health problems could explain some of the relationship between shift work and sleep quality.

High sleep latency, short sleep duration and low sleep efficiency are common signs of insomnia, which is defined by sleep initiation difficulty, sleep maintenance difficulty, early morning awakening or sense of nonrestorative sleep. Shift workers in our study scored significantly worse on these subscales compared to non-shift workers. Previous research has suggested that shift work is an independent risk factor for insomnia [44, 45].

### Mechanism of effect

The proposed mechanism of the observed increased odds of poor sleep quality associated with shift workers may be direct or indirect. A direct mechanism could be that the disruption of normal circadian rhythm leads to the lack of sleep. Circadian rhythm is synchronized around a 24-hour period and is regulated by the daily occurrences of light and darkness. Thus, it can be regarded that the sleep-wake cycle is the primary source of output for other functions that depend on circadian rhythm [46,47]. Any disturbance may reduce the release of melatonin and cortisol hormones, hence increasing the release of inflammatory markers which may contribute to metabolic syndrome or even cancer [48]. Inflammatory cytokines have been reported to be a significant contributor of disrupted sleep and poor sleep quality, and alterations in inflammatory cytokine levels have also been established in gastrointestinal disorders [49, 50]. In contrast, an indirect mechanism might be that shift workers have increased levels of secretion of serum gastrin (G) and group 1 pepsinogen (PG1) and it has been speculated that such increases may mediate the elevated risk for both gastric and duodenal ulcers in shift workers [5]. It is possible that an increase in both serum gastrin and pepsinogen levels also leads to sleep disturbance and ensuing sleepiness in shift workers [51, 52].

### Strengths and limitations

Our study has several strengths. First, it was based on a broad range of measures obtained from a large sample of workers from diverse occupational settings and ethnicities. Second, we used the PSQI instrument to assess sleep quality, which has been extensively validated in different populations, including Singapore, and found to be highly reliable and valid [53]. Moreover, we adjusted for several potential confounding factors, including concurrent use of caffeine, alcohol, and smoking status known to affect sleep quality. This strengthens the robustness of the relationship between shift work and sleep quality. Lastly, to our knowledge, this is the first study to evaluate the effects of shift work on quality of sleep using the PSQI instrument in a diverse population of workers in Singapore.

However, our study does have limitations. Owing to the nature of the cross-sectional design, we were not able to establish the direction of the relationship between shift work and sleep quality. In order to verify the direction of causality, it is necessary to show longitudinally that undertaking shift work is accompanied by an increase in odds in poor sleep quality. Shift work-related factors such as years working as shift workers, shift pattern (fixed or rotational), and intensity of work were not collected. Hence, such factors may be residual confounders. Moreover, individuals with poor sleep may be more motivated and engaged to participate in the study than those with good sleep. So the observed associations may be amplified due to self-selection bias of the study’s recruitment strategy. In our study, the assessment of the shift work among the workers is rather crude, thereby reducing the precision of any findings by failing to differentiate between individuals who work evening, night, early morning, or rotating shift. As this study was planned in a cross-sectional design, findings cannot be generalised to the wider population of workers with poor sleep in Singapore.

## Conclusion

Shift work was significantly and independently associated with increased odds of poor sleep quality in this sample of workers. The present findings should inform employment guidelines and help develop workplace health promotion interventions aimed at improving sleep quality among workers and ultimately lead to a healthier workforce.

## Supporting information

S1 Data (XLSX)

## Abbreviations

BMI: Body mass index
CI: Confidence interval
FFQ: Food Frequency Questionnaire
GHQ-12: General Health Questionnaire-12
GPAQ: Global Physical Activity Questionnaire
kg/m^2^: Kilogram per square metre
MET: Metabolic Equivalent
OR: Odds ratios
PSQI: Pittsburgh Sleep Quality Index
SD: Standard deviation
WHO: World Health Organization
